# Supplementary material for: Assessing Patient Satisfaction and the Need for Collaborative Treatment with Korean and Western Medicine
Source: Healthcare (Basel). 2024 Sep 23;12(18):1901. doi: 10.3390/healthcare12181901 (PMC11431174; doi:10.3390/healthcare12181901)
Supplement: Supplementary file 1 [file healthcare-12-01901-s001.zip › healthcare-3216877-supplementary.pdf]

Table S1. Participating institutions and study participants

Institution	Patients no.
Kyung Hee University Korean Medicine Hospital	30
Dongshin University Korean Medicine Hospital	30
Daegu Hanny University Hospital	19
Daejeon Jaseng Hospital of Korean Medicine	30
Mokpo Dongshin University Korean Oriental Hospital	30
Dong-Eui University Korean Medicine Hospital	16
Pusan National University Korean Medicine Hospital	9
Bucheon Jaseng Korean Medicine Hospital	3
Bundang Jaseng Hospital of Oriental Medicine	30
Wonkwang University Gwangju Medical Center	30
Wonkwang University Oriental Medicine Hospital, Jeonju	30
Wonkwang University Korean Medicine Hospital	6
Jaseng Hospital of Korean Medicine	13
Wonkwang University Jangheung Integrative Medical Hospital	30
Haeundae Jaseng Hospital of Korean Medicine	15
**Total study participants**	**321 patients**
